# The Role of 18F-Fluorodeoxyglucose (18F-FDG) Positron Emission Tomography/Computed Tomography (PET/CT) Values in the Detection of Bladder Carcinoma

**DOI:** 10.7759/cureus.105068

**Published:** 2026-03-11

**Authors:** Rika Yoshida, Takeshi Yoshizako, Shota Tanaka, Kazuya Okamura, Takashi Katsube, Hiroyuki Kuroda, Yoji Tsuchie, Yasushi Kaji

**Affiliations:** 1 Radiology, Faculty of Medicine, Shimane University, Izumo, JPN; 2 Radiology, Matsue Seikyo Hospital, Matsue, JPN; 3 Radiology, Shimane Prefectural Central Hospital, Izumo, JPN; 4 Radiology, Izumo City General Medical Center, Izumo, JPN

**Keywords:** 18f-fluoro-2-deoxy glucose (18f-fdg), bladder cancer, magnetic resonance imaging (mri), positron emission tomography-computed tomography (pet-ct), standardized uptake value (suv)

## Abstract

Background and objective

18F-fluorodeoxyglucose (FDG) is physiologically excreted into urine, which can obscure intravesical lesions and complicate the detection of bladder cancer on FDG positron emission tomography-computed tomography (PET/CT). This study aimed to evaluate the standardized uptake values (SUVs) of bladder cancer on FDG PET/CT and to assess the effects of reader experience and PET threshold modification on tumor detectability.

Methods

We retrospectively identified 15 patients with histologically confirmed bladder cancer who underwent MRI and PET/CT before surgery between April 2003 and December 2020. A nuclear medicine physician performed quantitative analyses using PET, CT, and PET/CT fusion images, as well as pelvic MRI, to measure the maximum standardized uptake value (SUVmax) of tumors and intravesical urine. Four radiologists independently reviewed axial PET, CT, and PET/CT fusion images. In the first session, readers were blinded to clinical information and used a fixed display threshold. In the second session, readers were informed of the presence of bladder cancer and permitted to adjust PET display thresholds.

Results

Among the 15 patients (median age: 75 years; 12 men), the median tumor size was 35 mm (range: 7-105 mm). Median SUVmax was 19.9 for tumors and 11.5 for urine. In blinded readings, less-experienced readers detected tumors in 60% of cases, whereas experienced readers detected 66.6%. After adjusting the threshold with increased cancer awareness, detection rates improved to 100% and 93.3%, respectively.

Conclusions

Bladder cancers demonstrated relatively high FDG uptake on PET/CT; however, lesion conspicuity varied depending on urinary FDG excretion. Quantitative assessment of tumor-to-urine metabolic contrast using the contrast rate (CR) and adjustment of PET display thresholds improved lesion detection in this cohort. These findings suggest that optimization of image interpretation strategies may enhance the diagnostic utility of PET/CT for intravesical tumor evaluation, although further studies with larger patient populations are required to validate these findings.

## Introduction

Studies have demonstrated the usefulness of 18F-fluorodeoxyglucose (18F-FDG) positron emission tomography/computed tomography (PET/CT) in the diagnosis, staging, and treatment response assessment of various malignancies [1-4]. In bladder cancer, FDG PET/CT has been primarily studied for lymph node staging and the detection of distant metastases, particularly in muscle-invasive disease [1-3,5,6]. However, its utility in evaluating primary bladder tumors remains debated.

One of the major limitations of FDG PET/CT in assessing bladder cancer is the physiological excretion of FDG into the urinary tract. Intense and heterogeneous urinary FDG accumulation within the bladder lumen often obscures lesions originating from the bladder wall, making reliable identification of intravesical tumors challenging [4]. Consequently, the detectability of bladder cancer on FDG PET/CT may depend not only on tumor metabolic activity but also on urinary FDG concentration and the approach to image interpretation. Although FDG PET/CT is not routinely used for the primary diagnosis of bladder tumors, the bladder is frequently included in the imaging field during PET/CT examinations performed for oncologic staging or evaluation of other conditions. Therefore, understanding how bladder tumors appear under physiological urinary FDG excretion may provide valuable insights for routine PET/CT interpretation.

In daily clinical practice, we occasionally encounter cases in which bladder tumors become detectable after modification of the PET display threshold, allowing better differentiation between tumor uptake and urinary activity. However, the impact of display threshold adjustments and reader experience on tumor detectability has not been systematically evaluated. Furthermore, quantitative comparisons of tumor FDG uptake with intravesical urinary activity have been insufficiently studied. In this context, the present study aimed to evaluate the standardized uptake value (SUV) of bladder cancer relative to intravesical urine on FDG PET/CT, to assess tumor detectability, and to investigate how reader experience and display threshold adjustments influence diagnostic performance.

## Materials and methods

Ethical approval and patient selection

This retrospective study was approved by the institutional review board, which waived the requirement for informed consent. After a search of the hospital information system, consecutive patients with histopathologically confirmed bladder cancer who had undergone both MRI and PET/CT before surgery (radical cystectomy or transurethral resection of bladder tumor [TUR-BT]) between April 2003 and December 2020 were identified and retrospectively evaluated.

All patients underwent PET/CT within three months of MRI. Patients who received neoadjuvant chemotherapy before imaging were excluded. Standard exclusion criteria for MRI (e.g., claustrophobia, pregnancy, or non-MRI-compatible pacemakers) were applied. Tumor staging was performed according to the Union for International Cancer Control (UICC) TNM classification, 8th edition [7]. Histopathological grading was assessed according to the World Health Organization (WHO) Classification of Tumours of the Urinary System and Male Genital Organs (2022 edition) [8].

PET/CT technique

Whole-body imaging was performed on a combined PET/CT scanner (True Point biograph 6, Siemens Medical Solutions). Whole-body CT and PET covered the region from head to mid-thigh. Neither oral nor intravenous contrast agents were administered during CT. The PET component had an axial view of 16.2 cm per bed position with an interslice spacing of 3.75 mm in one bed position, which provided an image with six or seven bed positions. The transaxial field of view and pixel size of the PET images reconstructed for fusion were 58.5 cm and 4.07 mm, respectively, with a matrix size of 168 × 168 and a spatial resolution of 4.5 mm. Patients were asked to drink 500 mL of water one hour before image acquisition and to then void just before image acquisition began to avoid urinary tract-associated artifacts. Urinary bladder catheterization was not performed.

After at least six hours of fasting, patients received an intravenous injection of FDG at 3.7 MBq/kg of body weight. Before the FDG injection, all patients underwent blood glucose level assessment, which found that none of the patients had a blood glucose level >200 mg/dL. Approximately 60 minutes after the FDG injection, a CT scan was performed after the patient had voided, which was immediately followed by whole-body emission PET with a three-min acquisition per bed position using a 3D acquisition mode. Attenuation-corrected PET images were reconstructed using an ordered-subset expectation maximization iterative reconstruction algorithm (eight subsets, three iterations). PET, CT, and fused PET/CT images were generated for review on a computer workstation XTREX VIEW (XTREX VIEW, J-MAC System, Sapporo, Japan).

MRI technique

MRI was performed with a 1.5-T scanner (Magnetom Lumina; Siemens, Erlangen, Germany) using a 32-element anterior torso phased-array coil coupled to an integrated posterior 20-element array in the tabletop. The MRI protocol comprised axial T2-weighted imaging (T2WI) in three orthogonal planes (transverse, sagittal, and coronal) and axial EPI-DWI (b = 0, 1000 s/mm²). Axial TSE-T2WI was performed using the following parameters: repetition time (TR)/echo time (TE), 3000-5000/90-100 ms; slice thickness, 4 mm; inter-slice gap, 1 mm; number of slices, 24; field of view, 21 cm; Turbo Factor, 11; and total scan duration of around three minutes.

Image analysis

Quantitative Analysis

One nuclear medicine physician with 10 years of experience in assessing FDG-PET/CT reviewed the PET, CT, and PET/CT image set, as well as the pelvic magnetic resonance images obtained within two months of the PET examination date, to measure SUV in patients suspected to have bladder cancer while referencing MRI findings (T2WI and DWI) and pathology results obtained from cystoscopy or surgery. Other imaging results or clinicopathologic findings other than the presence of bladder cancer were assessed using the Digital Imaging and Communications in Medicine (DICOM) Viewer (SDS DICOM Viewer; Techmatrix Ltd., Tokyo, Japan). For quantitative analysis of FDG uptake, a volume of interest (VOI) was created over the most intense area of FDG accumulation in the primary bladder cancer of each patient. SUVmax was used as the quantitative metric for all analyses. The VOI was carefully drawn to encompass only the bladder tumor while avoiding the surrounding urine.

The SUV of urine was calculated as follows: SUV = VOI radioactivity concentration (Bq/mL) / [injected dose (Bq) / patient’s weight (g)]. The SUVmax, which was defined as the highest SUV in the pixel with the highest count within the VOI, was measured and recorded for the focal areas of uptake. Given the heterogeneous distribution of urine within the bladder, urine activity was quantified by averaging measurements taken at two distinct intra-luminal locations, as well as at the anterior and posterior aspects of the bladder, with the patient in the supine position. All measurements were performed using the same workstation and analysis software to maintain consistency. Using the numerical values obtained, the contrast rate (CR) was calculated as (SUVmax of the cancer − SUVmax of urine in bladder) / (SUV max of the cancer + SUV max of urine in bladder), adapted from a previously reported contrast ratio calculation method [9].

Tumor Detection

Image interpretation was performed in accordance with established oncologic PET/CT reading principles [10]. Four radiologists independently reviewed axial PET, CT, PET/CT fusion images, and whole-body maximum intensity projection images. Two readers had one year of PET/CT experience, whereas the remaining two had 10 and 12 years of experience, respectively. In the first session, readers were blinded to clinical information and histopathologic findings and evaluated images using a fixed PET display threshold. All readers evaluated the images independently. In the second session, readers were informed that each patient had at least one bladder tumor and were allowed to freely adjust PET display thresholds. The sessions were separated by four weeks to minimize recall bias. The case order was randomized using Microsoft Excel. Pathological findings and MRI results served as the reference standard.

Statistical analysis

Data normality was assessed using the Kolmogorov-Smirnov test. Non-normally distributed variables were analyzed using the Wilcoxon signed-rank test. Detection differences between groups were evaluated using the Mann-Whitney U test. Detection outcomes in the reader study were recorded as binary variables (detected or not detected). Because the same readers evaluated the same cases in both reading sessions, detection rates between the first and second sessions were compared using McNemar’s test for paired categorical data. Statistical analyses were performed using SPSS version 23.0 (IBM Corp., Armonk, NY). A p-value <0.05 was considered statistically significant.

## Results

Patients

This study evaluated 15 patients (12 men, three women; median age: 75 years; range: 55-85 years) with bladder cancer.

Histopathologic findings

Table 1 summarizes the patients’ characteristics and histopathologic findings. All 15 patients had histopathologically confirmed bladder carcinoma, with the following histopathologic T category distribution: T1: six (40.0%); T2: two (13.3%); T3: five (33.3%); and T4: two (13.3%). The median maximum tumor diameter was 35 mm (range: 7-105 mm).

**Table 1:** Patient and tumor characteristics

Variables	Values
Sex	Total: 15 (M:F = 12:3)
Median age, years (range)	75 (55-85)
Tumor size, mm, median (range)	34.9 (7–105)
T stage (T1/2/3/4)	6/2/5/2
Histological type, n (%)	
Invasive urothelial carcinoma	6 (40%)
Non-invasive papillary urothelial carcinoma	6 (40%)
Squamous cell carcinoma	2 (13.3%)
Small cell neuroendocrine carcinoma	1 (6.7%)
Nuclear grade (high/low)	12 (80%)/3 (20%)

Histopathologic findings were as follows: non-invasive papillary urothelial carcinoma in six patients (40%), invasive urothelial carcinoma in six patients (40%), squamous cell carcinoma in two patients (13.3%), and small cell neuroendocrine carcinoma in one patient (6.7%). The pathological grade was high (G3 and G2) in 12 patients (80.0%) and low (G1) in three patients (20.0%).

Analysis of PET/CT findings

Quantitative Analysis

Table 2 details the results of the quantitative analysis.

**Table 2:** FDG uptake 18F-FDG: 18F-fluorodeoxyglucose; SUVmax: maximum standardized uptake value

Location	FDG uptake (SUVmax), median (range)
Bladder cancer	19.9 ± 9.9 (3.5–43.4)
Urine in the bladder	11.5 ± 12.9 (5.1–42.9)
Blood pool in the ascending aorta	1.8 ± 0.5 (1.3–3.0)
Background in the iliopsoas muscle	0.8 ± 0.1 (0.71–1.1)

The median SUVmax for bladder cancer was 19.9 ± 9.9 (range: 3.5-43.4), whereas the median SUVmax for urine in the bladder was 11.5 ± 12.9 (range: 5.1-42.9). The median SUVmax for blood pool in the ascending aorta was 1.8 ± 0.5 (range: 1.3-3.0), whereas the median SUVmax for the background in the iliopsoas muscle was 0.8 ± 0.1 (range: 0.71-1.1).

**Figure 1:**
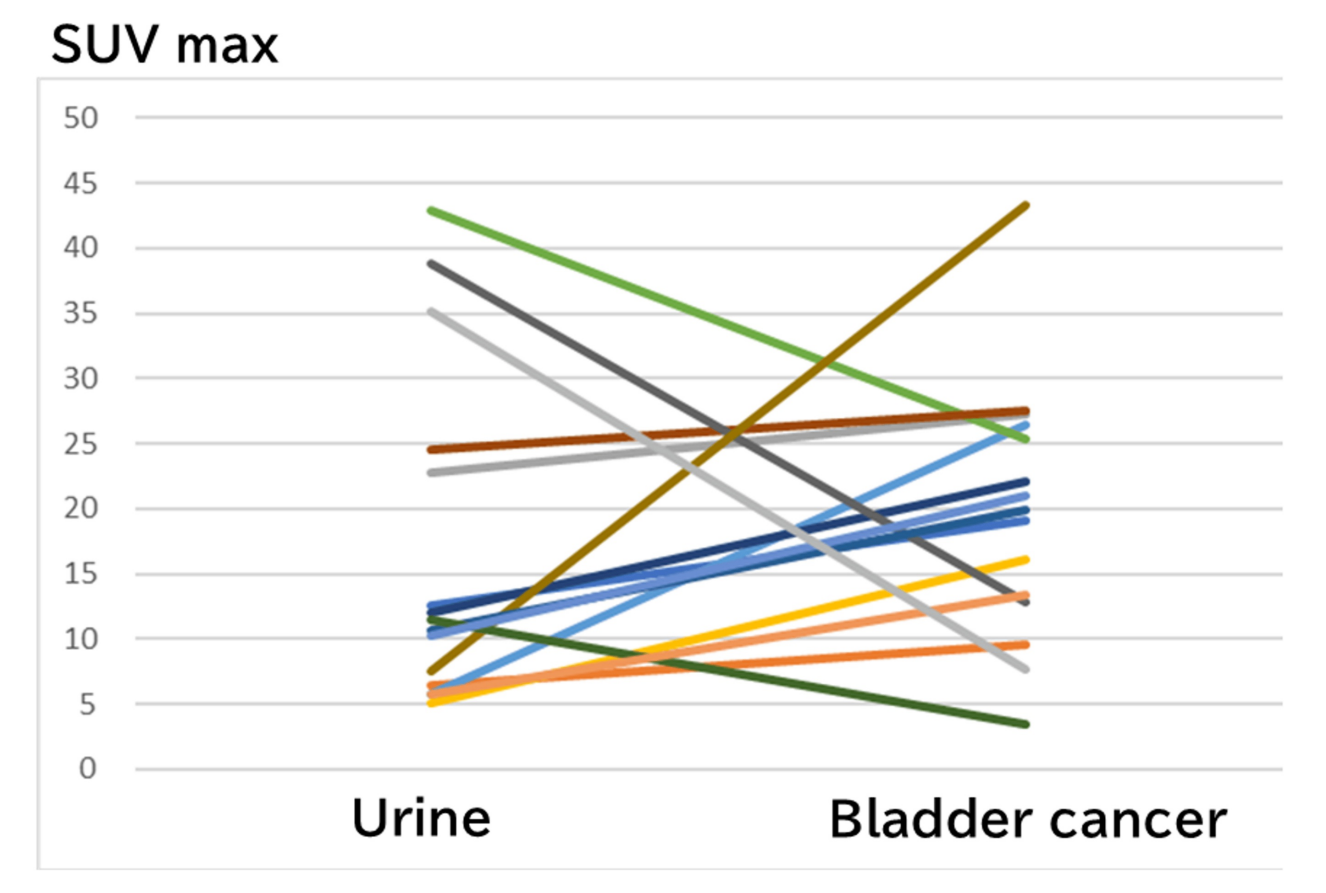
18F-FDG uptake for the urine and tumor in the bladder of each patient Despite variances in the accumulation of urine within the bladder of patients with bladder cancer, individual patients demonstrated some degree of accumulation 18F-FDG: 18F-fluorodeoxyglucose; SUVmax: maximum standardized uptake value

Figure 1 shows the FDG uptake for urine and tumor in the bladder of each patient.

The median CR was 0.21 ± 0.42 (range: −0.64-0.71). Bladder cancer showed high FDG uptake but may show positive or negative contrast.

Table 3 summarizes the FDG uptake according to pathological subtype.

**Table 3:** Tumor histology and 18F-FDG No significant difference in pathological subtypes or grades was observed according to FDG uptake. No significant difference in T stages was observed according to FDG uptake 18F-FDG: 18F-fluorodeoxyglucose; SUV: standardized uptake value

Pathology	No.	SUV mean	SUVmax
Invasive urothelial carcinoma	6	14.7	20.4
Papillary urothelial carcinoma	6	17.8	20.5
Squamous cell carcinoma	2	16.1	20.4
Small cell neuroendocrine carcinoma	1	6.7	6.7

No statistically significant difference in T stage and extent of FDG uptake was observed according to pathological subtype. Moreover, no significant difference in the histopathological results/grade/subtypes and the extent of FDG uptake was noted according to pathological subtype.

Tumor detection

In the initial reading session, readers 1 and 2 each had a tumor detection rate of 60.0%, whereas readers 3 and 4 both achieved 66.7%. Following the adjustment of the PET display thresholds during the second reading session, detection rates improved for all readers. Detailed results are presented in Table 4.

**Table 4:** Tumor detection details Regarding the reading results, 0 indicates that the reader could not identify the lesion, whereas 1 indicates they successfully identified the same SUVmax: maximum standardized uptake value

	Bladder cancer			Urine	Reader 1		Reader 2		Reader 3		Reader 4	
Case	Size (mm)	Location	SUVmax	SUVmax	1st	2nd	1st	2nd	1st	2nd	1st	2nd
1	23	Lateral	27.5	24.6	0	1	0	1	0	1	0	1
2	7	Posterior	19.12	12.5	0	1	0	1	0	1	1	1
3	35	Posterior	12.8	38.9	0	1	0	1	0	1	0	0
4	75	Posterior	20.96	10.29	0	1	0	1	1	1	0	1
5	9	Anterior	22.1	12	0	1	0	1	0	1	0	1
6	34	Lateral	43.39	7.5	1	1	1	1	1	1	1	1
7	42	Lateral	19.9	10.7	1	1	1	1	1	1	1	1
8	40	Posterior	13.4	5.8	1	1	1	1	0	1	1	0
9	40	Posterior	26.4	5.8	1	1	1	1	1	1	1	1
10	105	Lateral	7.6	35.2	1	1	1	1	1	1	1	1
11	12	Lateral	25.3	42.9	0	1	0	1	1	1	1	0
12	36	Lateral	16.1	5.12	1	1	1	1	1	1	1	1
13	10	Posterior	9.6	6.5	1	1	1	1	1	1	1	1
14	35	Lateral	3.5	11.5	1	1	1	1	1	0	1	1
15	20	Posterior	27.2	22.8	1	1	1	1	1	1	0	0

Specifically, readers 1 and 2 achieved detection rates of 100%, whereas detection rates for readers 3 and 4 increased to 93.3% and 73.3%, respectively. Overall, tumor detection improved from 63.3% (38/60) in the first reading session to 91.7% (55/60) in the second session.

Paired categorical analysis demonstrated a significant improvement in tumor detection following PET display threshold adjustment (McNemar’s test, p<0.001). Additionally, Table 5 provides details regarding the detected and non-detected groups.

**Table 5:** Details for the detected and non-detected cases ^*^Significant difference between the two groups Regarding the reading results, 0 indicates that the reader could not identify the lesion, whereas 1 indicates that they successfully identified the same N/A: not available; CR: contrast rate; SUVmax: maximum standardized uptake value

	Detection	Reader 1		Reader 2		Reader 3		Reader 4	
		1st	2nd	1st	2nd	1st	2nd	1st	2nd
Number	0	6	0	6	0	5	1	5	4
	1	9	15	9	15	10	14	10	11
Mean size (mm)	0	24.8	N/A	24.8	N/A	22.8	34.9	32.4	37.8
	1	40.2	34.9	40.2	34.9	40.9	35.0	36.1	26.8
Mean SUVmax	0	21.3	N/A	21.3	N/A	19.0	3.5	22.1	19.7
	1	18.6	19.7	18.6	19.7	20.0	20.8	18.4	19.7
Mean SUVmax of urine	0	23.5	N/A	23.5	N/A	18.8	11.5	21.7	12.3
	1	12.3	16.8	12.3	16.8	15.8	17.2	14.4	27.6
Mean CR	0	0.023	N/A	0.023	N/A	0.09^*^	−0.533	0.055	−0.070
	1	0.184	0.120	0.184	0.120	0.135^*^	0.167	0.152	0.189

**Figure 2:**
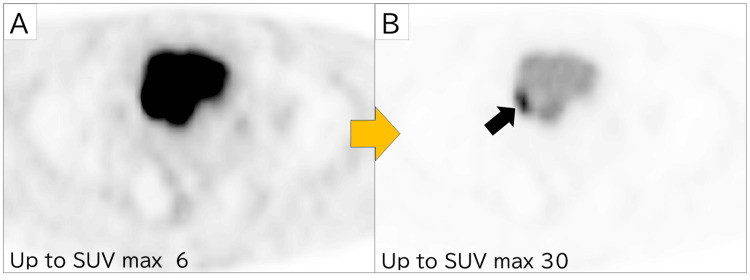
Representative PET/CT case for the staging of other diseases Imaging findings in a 57-year-old man with bladder cancer who had right ureteral invasion (high-grade squamous cell carcinoma). After changing the PET display condition from SUVmax 6 (A) to SUVmax 30 (B), the readers were able to identify a tumor on the right side of the bladder PET/CT: positron emission tomography-computed tomography; SUVmax: maximum standardized uptake value

During the initial interpretation, performed without clinical information and with fixed PET/CT threshold settings, tumors tended to be detectable after they had become large, had low urine SUVmax, and exhibited a high CR. This suggests that approximately 63% (38/60) of bladder tumors could be detected without modifying the imaging parameters when they were readily distinguishable from the surrounding urine. During the second image interpretation experiment, both readers 2 and 3 encountered false-positive cases. Specifically, reader 2 identified two such instances, whereas reader 3 found one. In all such cases, the readers mistakenly interpreted the normal accumulation of radioactive material at the right ureterovesical junction during urine excretion as indicative of a potential cancerous lesion. Representative images are provided in Figure 2.

## Discussion

PET/CT plays an established role in the evaluation of bladder cancer, particularly for nodal staging and detection of distant metastases in muscle-invasive disease [1,2,5,11-15]. However, its performance in detecting primary intravesical tumors remains limited because of the physiological urinary excretion of FDG [1-4]. Intense and heterogeneous FDG accumulation within the bladder lumen frequently obscures bladder wall lesions, thereby reducing lesion conspicuity and complicating interpretation. In the present study, bladder tumors demonstrated relatively high FDG uptake (median SUVmax: 19.9); however, tumor-to-urine contrast varied considerably, resulting in both positive and negative contrast patterns. This observation indicates that tumor detectability on PET/CT is influenced not only by absolute metabolic activity but also by the relative difference between tumor uptake and urinary FDG activity. The calculated CR provided a quantitative representation of this relationship and was associated with detectability in selected readers, underscoring the importance of tumor-to-background contrast in intravesical assessment.

Our reader experiment demonstrated that lesion detection rates improved substantially when readers were informed of the presence of the tumor and allowed to modify PET display thresholds. These findings suggest that interpretive strategy and display parameter manipulation significantly affect diagnostic performance. Although PET/CT interpretation is typically standardized, dynamic adjustment of SUV window levels may enhance visualization of subtle intravesical lesions, particularly in cases with moderate tumor-to-urine contrast. Established oncologic PET/CT reading principles emphasize the importance of contextual image assessment and awareness of physiological uptake patterns [10]; however, the specific impact of SUV threshold adjustment on bladder tumor detection has not previously been systematically evaluated.

Interestingly, less experienced readers achieved detection rates comparable to or exceeding those of experienced readers after threshold adjustment. One possible explanation is that experienced readers may apply stricter internal thresholds to avoid false-positive findings, particularly in regions prone to physiological FDG accumulation, such as the ureterovesical junction. Indeed, false-positive interpretations during the second session were primarily related to physiological uptake at this location. These findings highlight the need for balanced training strategies that improve sensitivity while maintaining specificity.

Recent studies and meta-analyses have suggested that PET/MRI may provide improved locoregional staging performance compared with PET/CT, particularly for primary tumor evaluation [14-16]. Nevertheless, PET/CT remains widely available and routinely used in clinical practice. Optimization of interpretation strategies for PET/CT, therefore, remains clinically relevant. Although FDG PET/CT is primarily used for staging in bladder cancer and is not intended to replace standard diagnostic approaches for primary tumor evaluation, our findings suggest that information about the primary tumor can also be obtained from these examinations. Appropriate adjustment of PET display thresholds and awareness of tumor-to-urine metabolic contrast may improve the detection of intravesical lesions.

Our findings indicate that simple, non-invasive modifications such as display threshold adjustment and awareness of tumor-to-urine contrast relationships may enhance diagnostic yield without requiring additional imaging protocols. Although FDG PET/CT is not intended to replace established diagnostic approaches such as cystoscopy, CT, or MRI for the evaluation of primary bladder tumors, the bladder is frequently included in the imaging field during routine PET/CT examinations performed for oncologic staging or for the evaluation of other malignancies. Under these circumstances, bladder tumors may occasionally be encountered incidentally. Therefore, understanding the imaging characteristics of intravesical tumors in the presence of physiological urinary FDG excretion and recognizing strategies that improve lesion conspicuity may contribute to more accurate interpretation of routine PET/CT examinations in clinical practice.

Limitations

Several limitations of this study should be acknowledged. Firstly, this was a retrospective, single-center study with a small sample size, which may limit generalizability. These factors may also have reduced statistical power. The relatively small sample size may also have limited the statistical power to detect differences in FDG uptake among histological subtypes. Therefore, the absence of statistically significant differences between tumor subtypes should be interpreted with caution, and larger studies are warranted to clarify potential metabolic differences among pathological subtypes. Second, PET/CT was performed after voiding in all patients, and no standardized bladder distension protocol was used. Third, negative control cases were not included, limiting the assessment of specificity. Fourth, prior notification of tumor presence during the second reading session may have introduced confirmation bias. Finally, quantitative measurements were performed by a single nuclear medicine physician, which may limit reproducibility. Despite these limitations, this study provides quantitative and interpretive insights into intravesical FDG uptake patterns and highlights the impact of image interpretation strategies on lesion detection.

## Conclusions

Bladder cancers demonstrated relatively high FDG uptake on PET/CT; however, lesion conspicuity varied depending on the degree of urinary FDG excretion. Quantitative evaluation using the CR highlights the importance of tumor-to-urine metabolic relationships in determining lesion detectability. Adjustment of PET display thresholds and awareness of tumor presence improved lesion detection in our reader experiment, emphasizing the importance of interpretation strategies in intravesical tumor evaluation. Although this study included a limited number of patients, these findings suggest that optimized image interpretation techniques may improve the practical diagnostic value of PET/CT for bladder tumor assessment without requiring additional imaging protocols.
